# General practitioners’ perceptions of compassionate communities: a qualitative study

**DOI:** 10.1186/s12904-020-00597-y

**Published:** 2020-07-06

**Authors:** E. Abbey, C. Craig, C. R. Mayland

**Affiliations:** 1 Department of Oncology and Metabolism, University of Sheffield, Sheffield, UK; 2 Faculty of Medicine, Dentistry and Health, University of Sheffield, Sheffield, UK; 3 Art and Design Research Centre, Sheffield Hallam University, Sheffield, UK; 4 Palliative Care Institute, University of Liverpool, Liverpool, UK

**Keywords:** Palliative care, Public health palliative care, Compassionate communities, General practice, General practitioners, Qualitative study, Public health

## Abstract

**Background:**

General Practitioners (GPs) face challenges when providing palliative care, including an ageing, multimorbid population, and falling GP numbers. A ‘public health palliative care’ approach, defined as “working with communities to improve people’s experience of death, dying and bereavement”, is gaining momentum. ‘Compassionate communities’ is one example, with a focus on linking professional health carers with supportive community networks. Primary care is central to the approach, which has been incorporated into United Kingdom GP palliative care guidance. No research to date, however, has investigated GP perspectives of these approaches. Our aim, therefore, was to explore GP perceptions of a public health approach to palliative care, and compassionate communities.

**Methods:**

GPs working in the United Kingdom were recruited through university teaching and research networks using snowball sampling. Purposive sampling ensured wide representation of gender, level of experience and practice populations. Semi-structured, digitally audio-recorded interviews were conducted with nine GPs. Interviews were transcribed *verbatim*, and thematic analysis was undertaken, informed by a qualitative descriptive methodology. Interviews continued until data saturation was reached.

**Results:**

Most participants were unfamiliar with the term ‘compassionate communities’, but recognised examples within their practice. Three major themes with seven subthemes were identified: 1) Perceived potential of compassionate communities, including: ‘maximising use of existing community services’; ‘influencing health outside of healthcare’; and ‘combatting taboo’, 2) Perceived challenges of compassionate communities, including: ‘patient safety’; ‘limited capacity of the community’; ‘limited capacity of general practice’, and ‘applicability of public health to palliative care’, and 3) The role of the GP in compassionate communities.

**Conclusions:**

GPs recognised the importance of the wider community in caring for palliative care patients, however most were unfamiliar with the compassionate community approach. Participants held differing views regarding the application of the model, and the position of general practice within this. Further research into the approach’s practical implementation, and exploring the views of other key stakeholders, would help establish the feasibility of compassionate communities in practice, and guide its future application.

## Background

Palliative care aims to improve the quality of life of patients and their families facing life-threatening illness by early identification and treatment of physical, psychosocial and spiritual needs [1]. Demand for palliative care is rising in line with an aging population and increasing multimorbidity [2, 3]. By 2040, 75% of people nearing end-of-life may benefit from palliative care [4]. In the United Kingdom (UK), complex palliative care needs are met by specialist teams, including palliative care consultants and specialist nurses, often based in hospices or hospitals [4]. However, palliative care is also frequently provided in the community by general practitioners (GPs) and district nurses [5]. General practitioners are well placed for this role given their broad clinical expertise, strong patient relationships, capacity for home visits and position as care coordinator [6]. They face barriers, however, to providing high quality palliative care, due to inadequate training in the specialty, ineffective communication between primary and secondary care, part-time work patterns, and increasing workload [7, 8]. GP workload is known to be high, with yearly rises in consultation numbers since 2008, compounded by increasingly complex cases [9, 10]. Whilst the number of GP trainees in the UK rose in 2019, the number of fully qualified GPs fell, and almost a third of GPs have stated they plan to leave direct patient care by 2022 [11, 12]. Strain on secondary care services also remains high, with a 4.9% increase in admissions to major accident and emergency departments in the last five years [13]. It follows that meeting increasing demands for palliative care within the current model of service provision will be challenging.

One model of palliative care which has been gaining momentum in recent years is a ‘public health palliative care’ approach. The approach is based on the principles of the 1980s ‘new public health’ movement; prevention, early intervention, and a view that health is everyone’s responsibility [14]. As part of this movement, multiple channels including schools, workplaces, government policy and the media, all became involved in spreading key health messages, for example relating to alcohol, smoking, and sexual health [15]. ‘Public health palliative care’ can broadly be seen as the application of these ‘new public health’ principles to palliative and end-of-life care [15, 16]. This is achieved by moving away from solely traditional, service-orientated provision of palliative care towards wider policies and community engagement to improve experience of death, dying and loss [16]. The movement led to the establishment of Public Health Palliative Care International, and Public Health Palliative Care UK, in 2015 [16]. In reality, several related terms have developed under the ‘public health palliative care’ umbrella, such as ‘health promoting palliative care’, ‘compassionate cities’ and ‘compassionate communities’ [16]. Indeed the lack of clarity in the terminology relating to this rapidly evolving field has been previously noted [16]. ‘Compassionate communities’ is one intervention which has gained particular traction. It “encourages communities to support people and their families who are dying or living with loss”, by linking professional healthcare to naturally occurring supportive networks already existing in communities, such as social groups, religious organisations and befriending services [17]. Examples have been established in Australia, India, and Ireland, amongst others [15].

Potential benefits of various social factors on health have already been documented. For example, the quality of social relationships have been found to impact mortality to the same degree as many ‘traditional’ risk factors [16]. In addition, the compassionate communities model, when implemented in Frome, UK, saw emergency hospital admissions across the town’s population reduce by around a third [18, 19]. Compassionate communities places primary care at the centre of change [20]. No research, however, has yet explored GP opinions of these new models, and the potential role of the GP within them. The aim of this study was therefore to explore GP experiences of providing community palliative care, the barriers and facilitators of this, and their perceptions of a public health palliative care approach and compassionate communities. This paper will focus on perceptions of compassionate communities and a public health palliative care approach.

## Methods

### Participant recruitment

GPs within University of Sheffield teaching and research networks were invited to participate in this study via email. Study materials provided background on the research team and aims of the research. Additional potential participants were identified via snowball sampling, from professional contacts of original participants. A purposive sampling approach was used when contacting new potential participants to generate a maximum variety sample in terms of gender, years of experience, and Index of Multiple Deprivation (IMD) score of the participant’s practice area. The IMD is an official measure of relative deprivation in England, based on multiple domains including income, employment, crime and education [21].

### Informed consent

Informed consent was obtained from all study participants. Verbal consent was obtained prior to interview. Written consent was provided at the time of the interview, either in person or via email in the case of telephone interviews. One participant (09) provided verbal consent for a telephone interview, however did not return a written consent form. Therefore, data from this interview was not included in the analysis.

### Data collection

Semi-structured, digitally audio-recorded interviews were conducted between 1st April and 30th June 2019. Interviews were face-to-face wherever possible, on University of Sheffield premises or at the participant’s home. Telephone interviews were undertaken where face-to-face interviews were not possible. All interviews were conducted, recorded and transcribed *verbatim* by EA, an academic junior doctor. EA did not have existing, established relationships with participants prior to interview. Mean interview duration was 30 min, ranging between 20 and 38 min. Field notes were taken immediately after each interview to capture researcher initial impressions.

An interview schedule was used for consistency across interviews, and to enable collection of comprehensive, open-ended data (Additional file 1) [22, 23]. Development of the interview schedule was based on a literature review and discussions with senior researchers (CM and CC) with significant experience in palliative care and qualitative research. It encouraged exploration of each participant’s current role in palliative care provision, the barriers and facilitators to community palliative care provision, and knowledge and perceptions of compassionate communities and public health palliative care. The interview schedule was pilot-tested prior to starting data collection.

After interviewing nine participants, with the last two interviews presenting no new concepts, we determined data saturation was reached and stopped interviewing.

### Analysis

Thematic analysis was chosen as it is a flexible approach which can provide “a rich and detailed, yet complex account of the data” [24]. These characteristics suit the broad, exploratory nature of the study. The approach involves six stages; familiarisation with the data, generating initial codes, searching for themes, reviewing, defining and naming these themes, and finally producing a report [24]. Qualitative descriptive methodology informed the analysis [25]. Analysis was essentialist, meaning it reported “experiences, meaning and the reality of participants” [24]. An inductive approach meant analysis was led by the data, without predetermined themes [24]. Interview transcripts were read several times (EA) to enable familiarisation with the data, and initial impressions were recorded. Preliminary codes were identified from transcripts. Four interview transcripts were independently analysed and coded by a second senior researcher (CM or CC). Further refinement of themes occurred throughout the analysis, and final themes were determined by consensus of the three researchers.

Techniques to enhance qualitative rigour included independent analysis of initial interviews and triangulation between researchers [26]. Outlying views were considered during analysis, to avoid making unwarranted claims about the data [26]. In the presentation of results, themes are illustrated by data extracts, and quotes have been selected from a spread of participants. Quantifying statements help to clarify patterns, whilst maintaining the focus on identifying meaning in participant experiences [26]. Participant characteristics have been presented to provide context and enable interpretation of the results [27].

## Results

Twenty five potential participants were contacted via email. Fourteen did not respond, one responded but later dropped out due to time constraints, and ten agreed to be interviewed. The majority of participants were female. Participants had a wide range of experience and represented practices from differing deprivation levels by IMD score (Table 1). IMD is presented as a numerical score, with scores ordered into quintiles. The first quintile represents the least deprived areas, and the fifth quintile represents the most deprived [28]. Participants were asked to declare any specialist interest in palliative care during interviews, for example completion of relevant post-graduate qualifications, or leading palliative care within their practice. Two participants declared a current interest in palliative care. One participant, who had worked as a GP for 22 years, recalled a specialist interest in palliative care early in their career, but had not pursued this for many years.

**Table 1:** Participant characteristics

	Gender	Years qualified as a GP	Current practice IMD score	Palliative care interest?	Region in the UK	Interview method
**01**	Female	29	58.55 (5th quintile)	No	S. Yorks	Face-to-face
**02**	Male	32	62.94 (5th quintile)	No	S. Yorks	Face-to-face
**03**	Female	10	20.00 (3rd quintile)	No	S. Yorks	Face-to-face
**04**	Male	25	50.86 (5th quintile)	No	S. Yorks	Face-to-face
**05**	Female	9	14.38 (3rd quintile)	No	S. Yorks	Face-to-face
**06**	Female	12	Locum	Yes	S. Yorks (previously West midlands)	Face-to-face
**07**	Female	14	10.95 (2nd quintile)	Yes	Surrey	Telephone
**08**	Female	13	Locum	No	S. Yorks	Face-to-face
**10**	Male	22	44.18 (5th quintile)	Previous	S. Yorks	Face-to-face

IMD, Index of Multiple Deprivation; S. Yorks, South Yorkshire

Three major themes, with additional subthemes, were identified in the analysis; 1) ‘perceived potential of compassionate communities’ with the subthemes ‘maximising use of existing services’, ‘influencing health outside of healthcare’ and, ‘combatting taboo’; 2) ‘perceived challenges of compassionate communities’ with the subthemes ‘patient safety’, ‘limited capacity of the community’, ‘limited capacity of general practice’, and ‘applicability of public health to palliative care’; and 3) ‘the role of the GP in compassionate communities’.

### Perceived potential of compassionate communities

#### Maximising use of existing community services

Participants frequently described services within their practice communities which could benefit palliative care patients. Examples included hospice-led day centres, soup and cake services, luncheon clubs, sporting memories groups, church groups, befriending services and other charitable organisations. They felt that patients, however, were unaware of the extent of support available outside general practice:

*“the majority of people do not know that … there are these different, erm, social groups that happen, erm, that are busy supporting people in their community.”* (GP 07).

Participants identified a ‘missed opportunity’ to raise awareness of these resources and incorporate them into medical practice. Many admitted that they rarely utilise these services in a palliative care context, and agreed that compassionate communities would offer one way to increase use of these networks in this patient group:

*“It makes perfect sense, doesn’t it … if we could activate what was already there in the community then it, it makes sense.”* (GP 03).

#### Influencing health outside of healthcare

Several participants recognised the influence of factors outside of formal healthcare on patient wellbeing:

*“I very, very much believe that health exists largely outside of medicine and healthcare … wellbeing and health is so much supported by our ability to interact with other people and feel like there’s meaning in our life and feel belonging to a group of people”* (GP 08).

The value of family support was recognised, despite increasing fragmentation of family units making this more difficult. Some GPs felt wider community members were unaware of the valuable contributions they could make to patients with terminal disease:

*“I would say all members of the practice team are important and all members of your, sort of, social community are important too, but they may not be aware of their role”* (GP 07).

Compassionate communities was seen as a way to foster an atmosphere of local support outside of the GP surgery, particularly for patients who might not benefit from more formal measures:

*“the socially isolated, living alone, erm, groups that are, or bedbound and very unwell … they do not want to go out to some very nicely formed groups that are being done on a Tuesday … more … your neighbours popping around, or getting a telephone call from your GP to see how you’re doing, or it could be an outreach person, erm, you know, does their daughter call on a regular basis? … do people say hello in the supermarket once a week when they go because that’s their only opportunity to get out? Erm, you know, does the hairdresser come and, can they come and visit? You know, all these little things that make up what your network is”* (GP 07).

#### Combatting taboo

Participants frequently perceived a societal taboo around death and dying, amongst the public, health care professionals (HCPs), politicians and the media. Living in a ‘death-defying’ society with advanced medical treatments was seen as further limiting exposure to death, and discouraging conversations about dying and advance care planning:

*“It’s a public health issue … People think of dying as a medical issue, and it’s not (laughs) it’s not a medical issue, it’s a natural issue and it happens to everyone … people aren’t talking about it, and I think that’s the major issue now … There’s no point educating all the health professionals to have conversations about dying when society’s not prepared to have them”* (GP 08).

Several participants mentioned the Death Café initiative. Death Cafés are free events where attendees are encouraged to discuss death, dying and loss in an informal, supportive environment, thus increasing awareness of these issues within society [29]. Participants believed they work to encourage conversation, but remain small-scale with potential for further development:

*“Death cafés … I think that’s a movement that, presumably would be part of the compassionate communities, but that really could be expanded more.”* (GP 04).

### Perceived challenges of compassionate communities

#### Patient safety

Several GPs were concerned that vulnerable patients could be put at risk through compassionate communities, and emphasised the measures required to minimise this:

*“there’d be the safety and security issues in terms of who was coming into houses and the appropriate, kind of, vetting of who that, who those people were. If there was any, sort of, money exchanging hands … there’d need to be, sort of, an infrastructure there”* (GP 03).

Similarly, several participants highlighted a need for training and support for individuals involved in compassionate communities, but who did not have a medical or social care background. Even with training, one GP pointed out the limits to the care which communities can safely provide:

*“I think at the end of life it does become quite complex, and I don’t think it’s necessarily something that I think voluntary organisations could support”* (GP 08).

#### Limited capacity of the community

GPs highlighted that communities’ abilities to care for their members are affected by a wider social and political picture, and have been limited by the current climate of austerity:

“*… you have to look at socioeconomic factors, and what are the drivers of community, of the ability of communities, to be responsive to and integrate with third sector charity providers, community volunteers etc etc … the way poorer communities have been made more poor has actually acted to put a huge strain on community resources … communities would be able to do more, and would be a greater resource, but … you need to have action and policies that value investment in communities”* (GP 01).

Participants acknowledged the limited time and resources available to charitable, public health and social care sectors, and were wary of increasing the responsibilities of these services. A number of participants viewed compassionate communities simply as a cost saving measure:

*“we can’t rely on these other charitable organisations to do things that should actually be properly funded elsewhere.”* (GP 05).

Furthermore, GPs were concerned that compassionate communities would not provide a stable source of care:

*“you’re setting up a service in which a whole, sort of, limb, if you like, of the service hasn’t got any kind of recurrent or stable resource.”* (GP 08).

#### Limited capacity of general practice

All participants commented on a heavy GP workload, and stated themselves or their colleagues felt unable to take on more duties. The initial investment of time and resources needed to set up compassionate communities was seen as a barrier:

*“You’ve got workforce crisis in general practice ... trying to get people to do something that is intensive at the beginning, so it will bring benefits … but you’re not going to see them straight away, you’re asking people to invest an awful lot up front … that’s gonna fail.”* (GP 08).

GPs were aware of limitations of the current community palliative care provision, particularly poor integration between different elements of care, and saw this as a barrier to implementing change:

*“I’d have concerns about adding complexity to an already complex model that isn’t delivering good teamwork or good interdisciplinary care.”* (GP 01).

#### Applicability of public health to palliative care

A number of participants perceived a disconnect between palliative care and public health:

*“You know, libraries and sports centres and things like that is what you tend to think of when you think about council-provided things and public health, isn’t it? … you kind of think ‘well that, what role do they have there [palliative care]?’ I’m not quite sure I see that”* (GP 05).

Participants often focussed initially on end-of-life care, and did not feel the involvement of compassionate communities was appropriate at this stage. However, on further discussion, some suggested a potential role earlier in the course of illness:

*“advanced life limiting illness is probably not the time to discover your community … I think in the longer run then, you know, sort of, early palliative … then if there’s time to form new communities, yeah.”* (GP 02).

Overall, there was a sense that, whilst there might be some role for a public health approach to palliative care, this was secondary to traditional services:

*“I wouldn’t want that [compassionate communities] to be then a reason to then not fund proper (laughs) palliative care services.”* (GP 05).

### The role of the GP in compassionate communities

Participants differed in their perception of the GP’s potential role within compassionate communities. Although only two participants were familiar with the term, all highlighted elements of current practice which would fit within the model. One participant discussed ‘Community Support Workers’, who work with the council and GP surgeries to support patients, including by enabling them to locate and join local social activities [30]. Another participant discussed the Daffodil standards, a collection of statements and tools designed to help GP practices provide high quality end-of-life care [31]. Participants already engaged in social prescribing, a model of signposting and referral of primary care patients to community and voluntary services and interventions [32]. They stressed, however, that this was rarely in a palliative care context. Many saw their potential role in compassionate communities as an extension of this signposting:

*“the obvious answer would be to say as a sort of linchpin in the middle, you know, liaising, not actually doing the specialist work, making sure that they’re drawing on the, the, the, if you like, the advantages or the strengths of the various groups that you’re talking about”* (GP 04).

Some participants were positive about this involvement, citing GP surgeries as natural ‘hubs’ of communities and knowledge. However, others disagreed, arguing that compassionate communities fell outside their role, and ‘signposting’ can be fulfilled by other HCPs, such as social workers or palliative care nurses:

*“GPs shouldn’t really sit in it [compassionate communities] at all (laughs) because it’s a social issue … it’s generally a social issue … ideally we would be freed up to deal with the medical side of things”* (GP 03).

Participants were supportive of empowering patients to seek help independently of formal referrals:

*“It can’t be GPs who are gatekeepers … it needs to be something where people are aware ‘in my community, this is something I can do.’”* (GP 01).

GPs frequently suggested that compassionate communities must be *led* outside of general practice to be successful:

*“I just really feel that compassionate community should, maybe, really be owned by the community. And I don’t think it’s necessarily ok for us as a body of healthcare professionals to come along and start doing unto the community. (laughs) Do you know what I mean? I think it would be much more powerful if the community own it, and take it forward.”* (GP 06).

The balance and integration of medical and social care was frequently discussed, with support for the compassionate communities concept balanced alongside a wariness of over-medicalisation:

*“there is a* willingness *to, erm, to do stuff at community level … but I don’t know that we should medicalise that.”* (GP 01).

## Discussion

This is the first qualitative study to explore GP perceptions of compassionate communities and a public health approach to palliative care in the UK. Generally, GPs recognised the value of community involvement in palliative care but lacked familiarity with the concept and some expressed concerns about their potential role within this approach. Whilst they perceived specific benefits such as helping combat the societal ‘taboo’ about death and dying, they anticipated several barriers to the models’ implementation.

‘Compassionate communities’ is a relatively new palliative care initiative that is gaining traction [33]. In our study, the majority of participants recognised services in their current practice related to the model, including social prescribing and the introduction of the Daffodil standards [31, 34–36]. However, they were not overly familiar with ‘compassionate communities’ as a distinct term or approach to palliative care. This is a concern given the emphasis in the broader literature as to the central role that GPs have in the implementation of a public health palliative care approach [17, 20, 31]. If the model is to be expanded, further engagement and training of GPs is an important consideration, to provide a clearer understanding of compassionate communities, their aims, and how this can be achieved in practice.

This study describes a number of perceived benefits of a public health palliative care approach. Death has long been recognised as a taboo subject [37]. More recently, the importance of GPs breaking down this taboo in order to enable better end-of-life care has been highlighted [38]. GPs within this study recognised compassionate communities as one tool which would enable them to achieve this. Participants also recognised the ability for compassionate communities to enhance the wellbeing of palliative patients outside of formal healthcare. This is in keeping with previous research where increasing social connectivity through compassionate communities improved the wellbeing of palliative care patients by providing peer-support which could not be met by HCPs or relatives [39].

GPs also perceived potential challenges of implementing compassionate communities. Participants were concerned that general practice currently lacks the capacity to take on further responsibilities due to limited time and funding. This is reflective of an increasing GP workload [9, 10], and has been cited as a barrier to community palliative care previously [7, 8]. GPs also stated that the community lacks the capacity to assume further caring responsibilities. Limitations of social care and community resources are well known, and it is estimated that between 2010 and 2020 UK councils have lost 60p in every £1 of central government funding [40]. The negative impact of funding cuts on health has been previously documented [41]. In addition, participants in our study emphasised that community members engaging with patients would need adequate training and support. This seems pertinent, as previous studies have shown that non-medical nursing home staff often feel underprepared in caring for people with palliative care needs in the community, in some cases leading to poor care and inappropriate hospital admissions [42]. Participants also questioned the applicability of public health to palliative care, querying the types of public health services which might be helpful to patients, particularly at end-of-life. There is a paucity of literature on the practical application of a public health approach to palliative care, and evidence of its effectiveness. A review of public health palliative care initiatives would be helpful to generate a picture of current practice and guide future direction, and a scoping review is indeed planned [16]. Further insight into the practical implementations of compassionate communities from other key stakeholders would be beneficial. This would include learning from care givers, volunteers within community support services, palliative care patients and their family members. In particular, focusing on perceptions about the optimum way to support community-members from the user-perspective would be pertinent.

### Strengths and limitations

This study has a number of strengths, for example the use of purposive sampling, which is known to enable high-quality, detailed case descriptions, and to identify shared patterns across participants [43]. Secondly, face-to-face interviews were undertaken, which are regarded as superior for the generation of rich narrative data [44]. In addition, the interviewer was an academic junior doctor with an interest in palliative care, whose existing knowledge of the topic helped guide interviews and analysis. Finally, independent analysis and coding of four transcripts by researchers helped ensure robust analysis. There are, however, a number of limitations to the study, for example a risk of interviewer bias. Prior discussion with senior researchers (CM and CC) helped guide interview technique to mitigate this. Secondly, the sample size was relatively small, however rich data was collected from each participant, and interviews continued until data saturation [45]. Finally, initial participants were recruited from an academic setting, and from a limited geographical region. It may be that our findings are not representative of HCPs from other health care settings, and other regions of the UK, and so they must be applied with caution. It would be useful to extend this study into additional regions throughout the UK, or internationally.

## Conclusions

A public health approach to palliative care is gaining momentum in policy and practice. GPs perceived value in community involvement, however were often unfamiliar with compassionate communities and their role within the model. They also anticipated a number of difficulties in its practical implementation. These challenges need to be addressed as the approach is developed further, to ensure its safety and efficacy for patients, and its feasibility for GPs and other care providers. Further engagement with primary care, and additional perspectives of other key stakeholders, would be useful to provide this clarity, and to inform the application of these approaches in the future.

## Supplementary information

**Additional file 1.** Interview topic guide.

## Data Availability

The datasets generated and analysed during the current study are not publicly available due some potentially identifiable content which may compromise individual privacy. However an anonymised version is available from the corresponding author on reasonable request.

## Abbreviations

GP: General Practitioner
HCP: Health care professional
