# Supplementary material for: General practitioners’ perceptions of compassionate communities: a qualitative study
Source: BMC Palliat Care. 2020 Jul 6;19:97. doi: 10.1186/s12904-020-00597-y (PMC7339557; doi:10.1186/s12904-020-00597-y)
Supplement: Supplementary file 1 — Additional file 1. Interview topic guide. [file 12904_2020_597_MOESM1_ESM.docx]

**Additional file 1**

**Interview topic guide**

**Introduction -** Interviewer to introduce themselves and explain purpose of the interview. Explain expected duration of interview is ≤ 30 minutes.

**Background Information -** Participant invited to give some information about themselves; where they work, how long they have been qualified, the demographic of their patient population, any specialist interests including research and teaching?

**Current community palliative care**

- Tell me about how palliative care is provided in a primary care setting?
- What different members of the multidisciplinary team are involved in providing palliative care in the community? What are their roles?

**Benefits of the current system**

- Are there certain situations where community palliative care is particularly successful?

**Barriers in the current system**

- Are there certain situations where community palliative care is less successful?
- What are the barriers to providing high quality palliative care in the community?

**How these barriers might be overcome**

- Do you have any suggestions for how services could change to improve your ability to provide good palliative care?

**New approaches**

- What is your understanding of compassionate communities?

*If the participant is unaware of compassionate communities, the background and concept will be explained – trial in Frome and other areas of close integration of community services with primary care to support patients at high risk of unplanned admission.*

- What are your thoughts about this concept?
- Do you think it might be an alternative way of addressing community palliative care?

**Thank the participant and close.**
